# Supplementary figures and images for: Mechanisms of oat (Avena sativa L.) acclimation to phosphate deficiency
Source: PeerJ. 2017 Nov 1;5:e3989. doi: 10.7717/peerj.3989 (PMC5671117; doi:10.7717/peerj.3989)

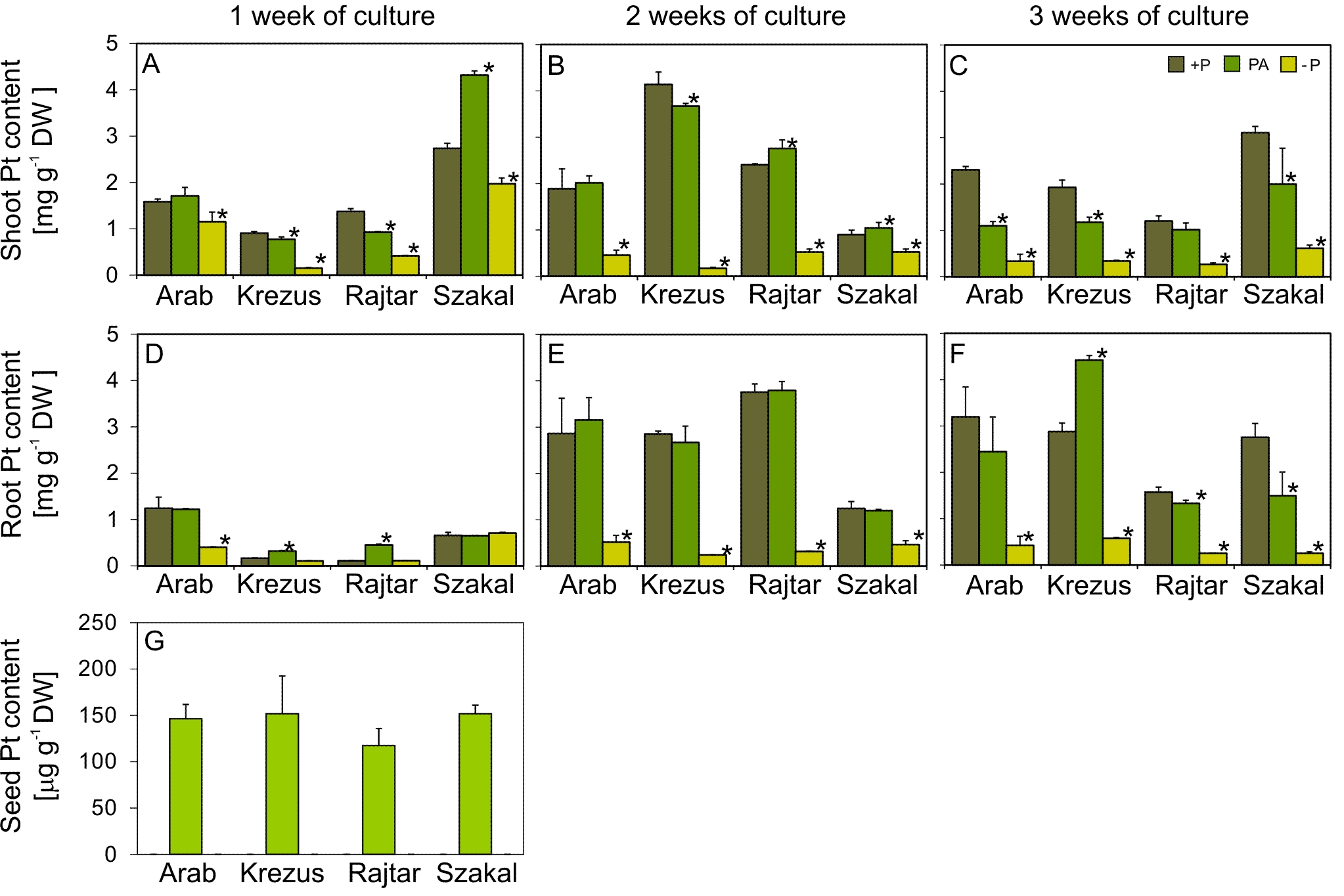

Supplement: Figure S1 — Oat varieties (Avena sativa L., Arab, Krezus, Rajtar and Szakal) were grown for 1, 2 and 3 weeks on complete nutrient medium (+P), medium with phytic acid (PA) and without phosphate (−P). *Differences statistically important at 0.05. [file peerj-05-3989-s001.png]

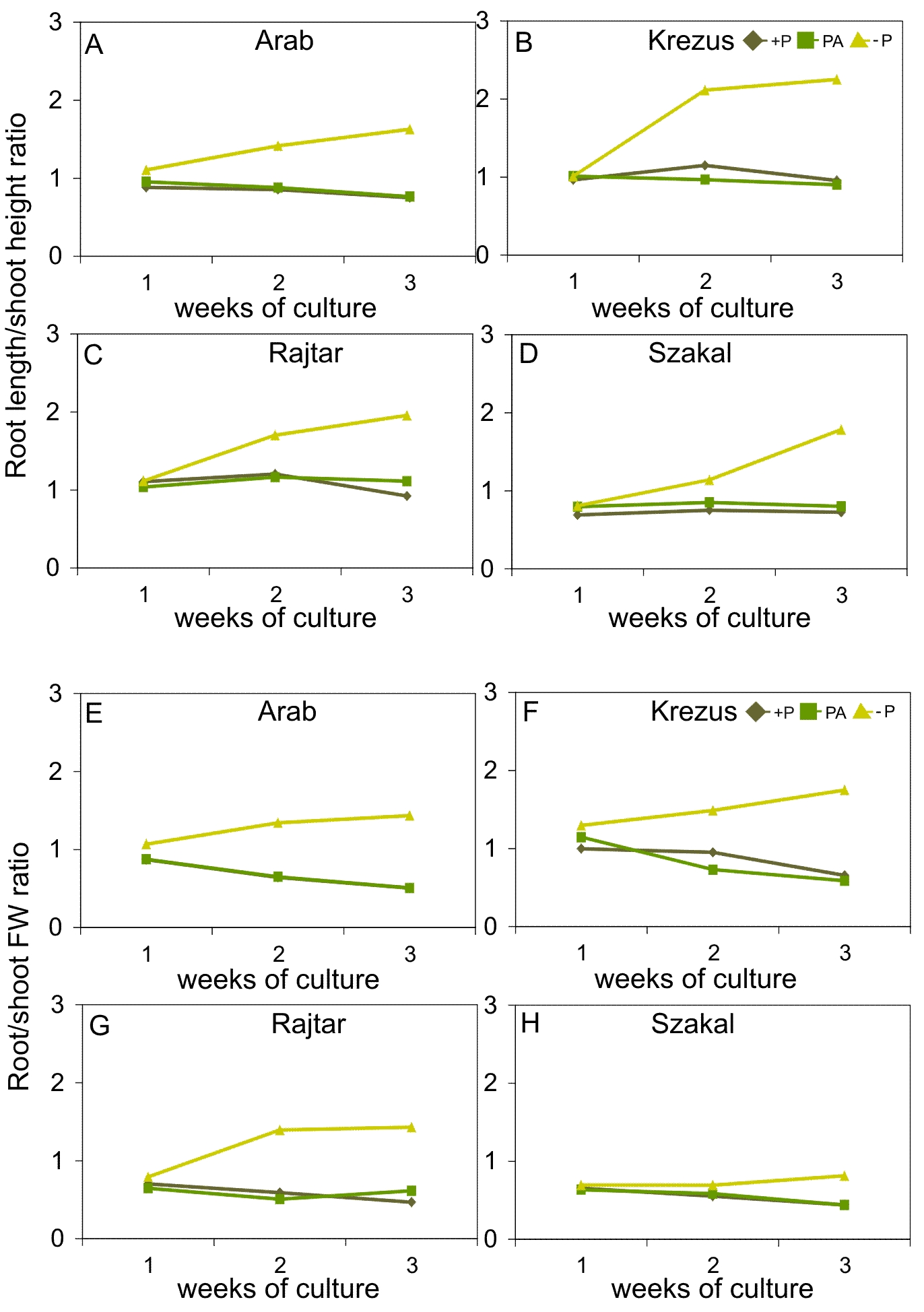

Supplement: Figure S2 — Root to shoot ratio of four oat varieties (Avena sativa L., Arab, Krezus, Rajtar and Szakal) grown for 1–3 weeks on complete nutrient medium (+P), medium with phytic acid (PA) and without phosphate (−P). [file peerj-05-3989-s002.png]

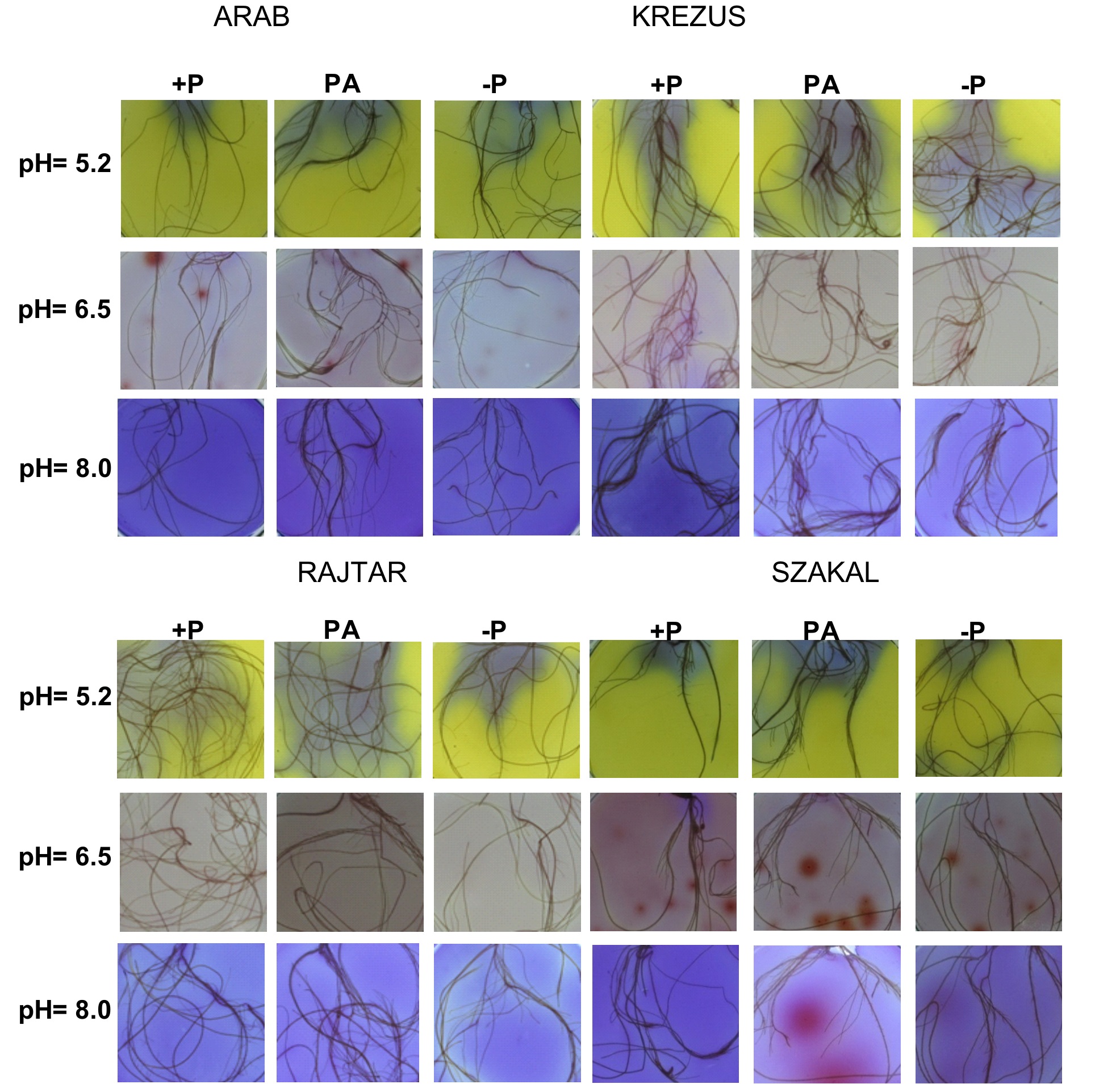

Supplement: Figure S3 — Modifications of rhizosphere pH (with bromocresol purple as indicator) of oat varieties roots (Avena sativa L., Arab, Krezus, Rajtar and Szakal) grown for one week on complete nutrient medium (+P), medium with phytic acid (PA) and without phosphate (−P). [file peerj-05-3989-s003.jpg]

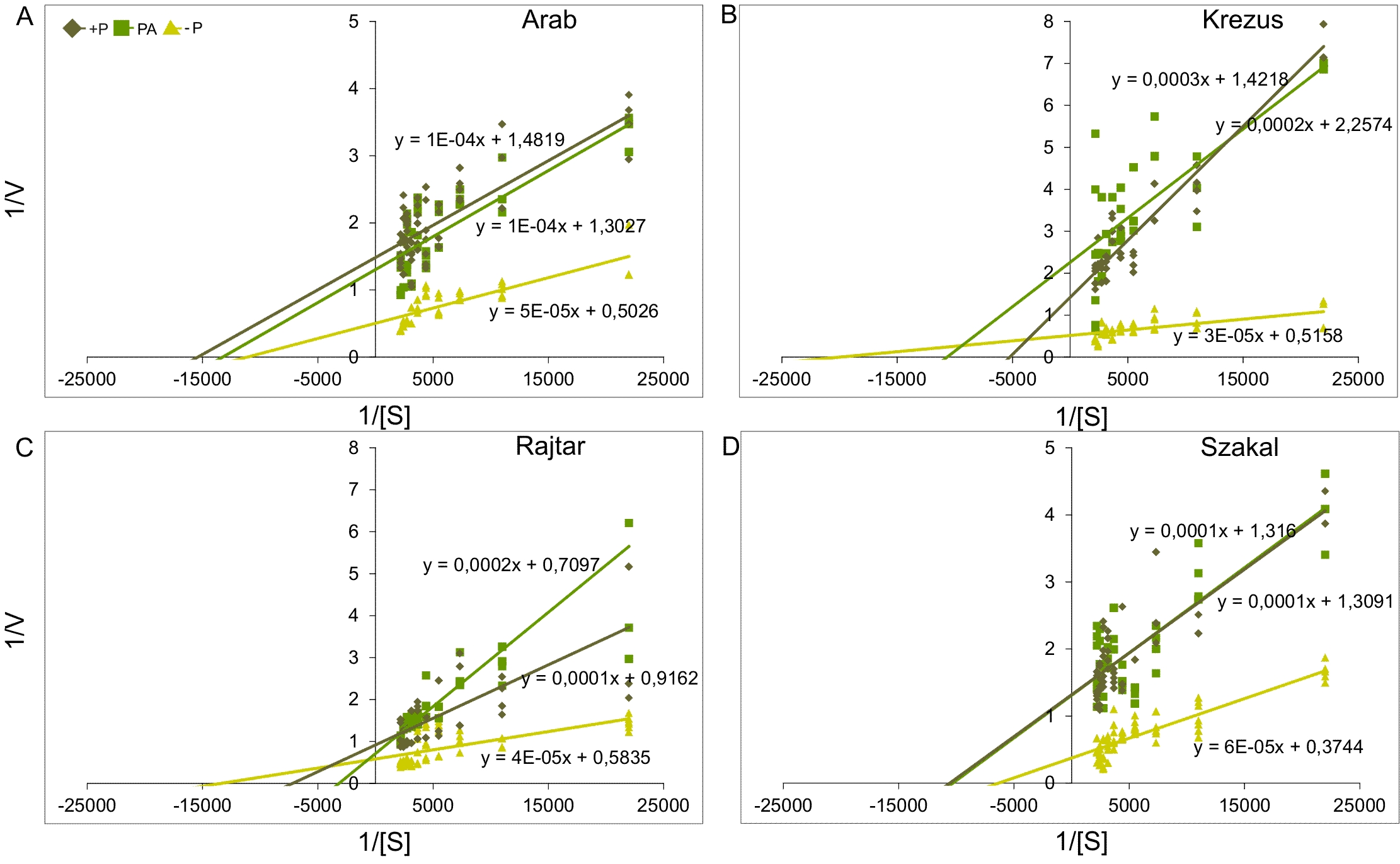

Supplement: Figure S4 — Extracellular APase activity kinetic assay, the Lineweaver–Burk plot and the Km and Vmax estimated for four oat varieties (Avena sativa L., Arab, Krezus, Rajtar and Szakal) grown for two weeks on complete nutrient medium (+P), medium with phytic acid (PA) and without phosphate (−P). [file peerj-05-3989-s004.png]
