# Supplementary material for: Mechanisms of oat (Avena sativa L.) acclimation to phosphate deficiency
Source: PeerJ. 2017 Nov 1;5:e3989. doi: 10.7717/peerj.3989 (PMC5671117; doi:10.7717/peerj.3989)
Supplement: Table S1 — Growth parameters of four oat (Avena sativa L.) varieties (Arab, Krezus, Rajtar and Szakal) grown for 2 weeks on complete nutrient medium (+P), medium with phytic acid (PA) and without phosphate (−P). *Differences statistically important at 0.05. [file peerj-05-3989-s005.docx]

**Table S1.** Growth parameters of four oat (*Avena sativa* L.) varieties (Arab, Krezus, Rajtar and Szakal) grown for 2 weeks on complete nutrient medium (+P), medium with phytic acid (PA) and without phosphate (-P). *Differences statistically important at 0.05.

| PARAMETER | ARAB | | |  | KREZUS | | |  | RAJTAR | | |  | SZAKAL | | |
| --- | --- | --- | --- | --- | --- | --- | --- | --- | --- | --- | --- | --- | --- | --- | --- |
|  | +P | PA | -P |  | +P | PA | -P |  | +P | PA | -P |  | +P | PA | -P |
| Shoot fresh mass [g] | 1.14 | 1.27 | 0.46* |  | 1.13 | 1.39 | 0.45* |  | 1.00 | 1.16 | 0.42* |  | 2.04 | 1.77 | 0.82* |
| Root fresh mass [g] | 0.73 | 0.83 | 0.62 |  | 1.07 | 1.02 | 0.68* |  | 0.59 | 0.59 | 0.58 |  | 1.13 | 1.03 | 0.57* |
| LAR [cm^2^ A g^-1^ W] | 20.8 | 17.6* | 17.9* |  | 22.1 | 18.9 | 14.4* |  | 24.2 | 22.8 | 26.3 |  | 20 | 20 | 24.4 |
| SWR [g WS g^-1^W] | 0.5 | 0.5 | 0.4* |  | 0.5 | 0.6 | 0.3* |  | 0.6 | 0.5* | 0.5* |  | 0.5 | 0.6 | 0.5 |
| ULR  [g^-1^∆dW cm^2^A^-1^ week^-1^] | 0.003 | 0.004 | 0.004 |  | 0.002 | 0.003 | 0.004* |  | 0.003 | 0.003 | 0.004 |  | 0.004 | 0.003 | 0.004 |
| RGR  [g ∆dW gW^-1^ week^-1^] | 0.06 | 0.06 | 0.08* |  | 0.04 | 0.05 | 0.05 |  | 0.07 | 0.06 | 0.08 |  | 0.08 | 0.08 | 0.06 |
| Root diameter [mm] | 0.9 | 0.9 | 0.7 |  | 0.9 | 0.9 | 0.7 |  | 0.7 | 0.7 | 0.7 |  | 1.1 | 0.9 | 0.7* |
| Root area [cm^2^] | 8.5 | 9.2 | 8.7 |  | 11.9 | 10.9 | 10.3* |  | 8.6 | 8.3 | 8.8 |  | 10.7 | 10.9 | 8.2* |

|  |
| --- |
|  |
