# Supplementary material for: Mechanisms of oat (Avena sativa L.) acclimation to phosphate deficiency
Source: PeerJ. 2017 Nov 1;5:e3989. doi: 10.7717/peerj.3989 (PMC5671117; doi:10.7717/peerj.3989)
Supplement: Table S2 — Chlorophyll and carotenoids contents in leaves of four oat (Avena sativa L.) varieties (Arab, Krezus, Rajtar and Szakal) grown for 1–3 weeks on complete nutrient medium (+P), medium with phytic acid (PA) and without phosphate (−P). *Differences statistically important at 0.05. [file peerj-05-3989-s006.docx]

**Table S2. Chlorophyll and carotenoids contents in leaves** of four oat (*Avena sativa* L.) varieties (Arab, Krezus, Rajtar and Szakal) grown for 1- 3 weeks on complete nutrient medium (+P), medium with phytic acid (PA) and without phosphate (-P). *Differences statistically important at 0.05.

| Pigments | ARAB | | |  | KREZUS | | |  | RAJTAR | | |  | SZAKAL | | |
| --- | --- | --- | --- | --- | --- | --- | --- | --- | --- | --- | --- | --- | --- | --- | --- |
|  | +P | F | -P |  | +P | F | -P |  | +P | F | -P |  | +P | F | -P |
|  | 1 week of culture | | | | | | | | | | | | | | |
| Chlorophyll *a+b*  [mg g^-1^FW] | 2.06 | 2.02 | 2.17 |  | 2.01 | 1.56 | 1.52* |  | 2.22 | 2.15 | 2.11 |  | 2.00 | 1.96 | 1.91 |
| Carotenoids  [mg g^-1^FW] | 0.51 | 0.53 | 0.41 |  | 0.71 | 0.60 | 0.61 |  | 0.70 | 0.73 | 0.75 |  | 0.77 | 0.74 | 0.69 |
|  | 2 weeks of culture | | | | | | | | | | | | | | |
| Chlorophyll *a+b*  [mg g^-1^FW] | 2.27 | 1.68* | 1.82* |  | 1.85 | 1.81 | 1.64 |  | 2.31 | 2.07 | 2.54 |  | 2.39 | 2.37 | 1.92 |
| Carotenoids  [mg g^-1^FW] | 0.70 | 0.66 | 0.67 |  | 0.62 | 0.62 | 0.52 |  | 0.79 | 0.80 | 0.85 |  | 0.79 | 0.74 | 0.69 |
|  | 3 weeks of culture | | | | | | | | | | | | | | |
| Chlorophyll *a+b*  [mg g^-1^FW] | 2.19 | 1.74* | 1.33* |  | 2.45 | 1.95 | 1.67 |  | 2.32 | 2.14 | 2.09 |  | 2.68 | 2.07 | 1.71 |
| Carotenoids  [mg g^-1^FW] | 0.43 | 0.48 | 0.31 |  | 0.65 | 0.67 | 0.55 |  | 0.79 | 0.83 | 0.74 |  | 0.91 | 0.82 | 0.86 |
|  |  |  |  |  |  |  |  |  |  |  |  |  |  |  |  |
